# Overcoming Resistance: FLT3 Inhibitors Past, Present, Future and the Challenge of Cure

**DOI:** 10.3390/cancers14174315

**Published:** 2022-09-02

**Authors:** Debora Capelli, Diego Menotti, Alessandro Fiorentini, Francesco Saraceni, Attilio Olivieri

**Affiliations:** Departement of Haematology, Azienda Ospedaliera Universitaria Ospedali Riuniti di Ancona, Via Conca 71, 60126 Ancona, Italy

**Keywords:** acute myeloid leukemia, FLT3 inhibitors, FLT3 mutation, overcoming resistance, mechanism of resistance, Midostaurin, Gilteritinib, Sorafenib, Quizartinib, Crenolanib

## Abstract

**Simple Summary:**

Despite the recent approval of some FLT3 inhibitors by drug regulatory agencies, treatment guidelines for FLT3-mutated AML still require allogeneic transplantation as a necessary procedure to treat the disease in first or second CR, due to the high relapse incidence related to the use of these drugs. The study of the heterogeneity of leukemogenesis and resistance mechanisms related to the use of FLT3 inhibitors, alone or in combination, represents one of the additional challenges in attempting to achieve the eradication of the mutated FLT3 leukemic clone. The analysis and knowledge of these pathways might drive future approach in this setting.

**Abstract:**

FLT3 ITD and TKD mutations occur in 20% and 10% of Acute Myeloid Leukemia (AML), respectively, and they represent the target of the first approved anti-leukemic therapies in the 2000s. Type I and type II FLT3 inhibitors (FLT3i) are active against FLT3 TKD/ITD and FLT3 ITD mutations alone respectively, but they still fail remissions in 30–40% of patients due to primary and secondary mechanisms of resistance, with variable relapse rate of 30–50%, influenced by NPM status and FLT3 allelic ratio. Mechanisms of resistance to FLT3i have recently been analyzed through NGS and single cell assays that have identified and elucidated the polyclonal nature of relapse in clinical and preclinical studies, summarized here. Knowledge of tumor escape pathways has helped in the identification of new targeted drugs to overcome resistance. Immunotherapy and combination or sequential use of BCL2 inhibitors and experimental drugs including aurora kinases, menin and JAK2 inhibitors will be the goal of present and future clinical trials, especially in patients with FLT3-mutated (FLT3mut) AML who are not eligible for allogeneic transplantation.

## 1. Introduction

Twenty-five to 30 percent of AMLs harbor FLT3 receptor mutations: 20–25% at the level of the juxtamembrane (JM) domain, recognized as internal tandem duplications (FLT3ITD) and distinguished, on the basis of the insertion site, in JM (70% of FLT3ITD) and TKD1 (30% of FLT3ITD) [1]; and 5–10% at the level of TK domain, especially at the D835 residue, known as tyrosine kinase domain mutations (FLT3TKD). The hyperactivation of chaperone proteins such as calnexin and HSP90 and the hypoglycosilation of the 130 KDa tyrosine kinase FLT3 protein cooperate in retention of FLT3 in the Golgi apparatus and endoplasmic reticulum, promoting leukemogenesis via the PIK/AKT/mTOR pathway and activation of STAT5 and Pim-1 (oncogenic serine-threonine kinase) downstream signaling [2,3,4,5].

The small amount of mutated FLT3 ITD proteins not hypoglycosilated remaining on the cell surface determines the alternative activation of PIK/AKT and MEK-ERK signaling.

**Figure 1:**
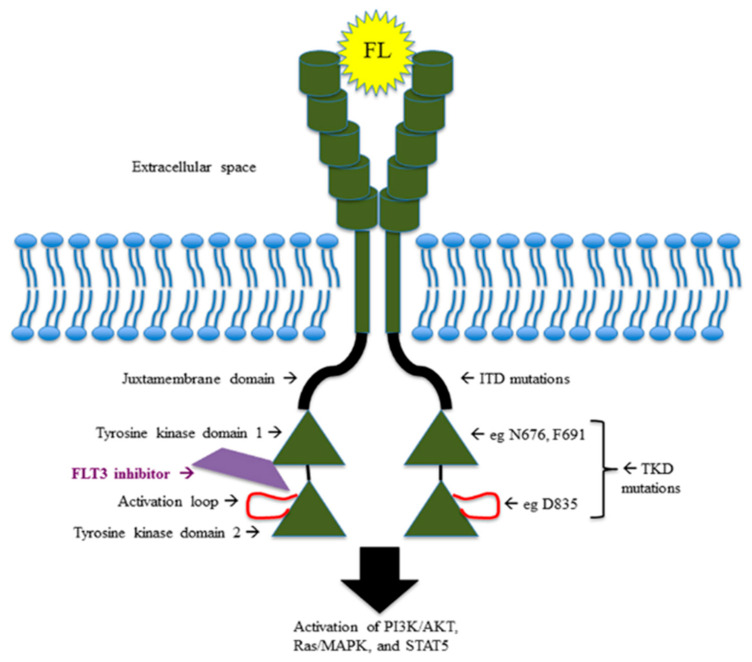
FLT3 pathway and FLT3 mutations.

FLT3TKD mutations have different downstream effects, resulting in blocking of differentiation, rather than stimulation of proliferation. For instance, FLT3ITD-mutated AML cells specifically determine STAT5 activation and p27 inhibition through the binding of their tyrosines residues 589 and 591 with Src family kinases (Lck, Hck, Fyn, Fgr, Lyn), which might explain the proliferative advantage of FLT3ITD-mutated AML over FLT3TKD-mutated AML [6,7]. Figure 1 shows mutations and pathway of FLT3 receptor.

FLT3i drugs differ in inhibition potency, activity on FLT3-ITD and TKD mutations, and specificity, and for these reasons they could have variable off-target toxicities [8].

Type I FLT3i (Lestaurtinib, Midostaurin, Gilteritinib, Crenolanib) are active against both FLT3-ITD and TKD mutations, because they interact with tyrosine kinase receptors in the active and inactive forms, while Type II FLT3i (Quizartinib and Sorafenib) are active only in the FLT3ITD forms because they have the binding site at the level of the hydrophobic region adjacent to the ATP-binding site, which is inaccessible when the receptor is in the active form. The availability of these drugs has dramatically changed the treatment guidelines for AML, supported by evidence of their efficacy with a molecularly guided approach.

FLT3i were some of the few target drugs approved in the 2000s; however, FLT3ITD AML still has an unfavorable outcome, especially when it occurs with high FLT3 allelic ratio in NPM wild type patients [9].

Incidence of relapse could be decreased by the use of second generation FLT3i, but NGS technologies and single-cell analysis have already demonstrated resistance pathways in cell cultures in vitro and in patients with FLT3ITDmut AML in vivo, and hematopoietic stem cell transplant (HSCT) is still necessary and recommended for curing the disease [10].

FLT3ITDmut AML, with low allelic ratio with NPM1 comutations, is classified in the 2017 ELN classification as a favorable risk disease; nevertheless, the predictive role of this association is still controversial [11,12].

The resistance could be driven by acquisition of other mutations with or without loss of FLT3 or by acquisition or switching to different FLT3 mutations. Moreover, bone marrow niche could induce resistance through the release of microenvironmental factors which compete for the same therapeutic target of FLT3i or promote blasts and leukemic stem cell survival. Indeed, upregulation of the target-downstream pathways, such as STAT5 or mTOR, may contribute to blocking FLT3i activity. Overexpression of proteins inhibiting the apoptosis and inhibition of drug metabolism by cytochromes expressed in stroma are some other mechanisms of leukemic escape [13,14]. Deeper knowledge of these topics could guide the future choice of novel target drug combinations to finally improve the cure of the disease. In this review, we focus on FLT3mut AML, briefly reviewing the clinical trials that have determined the indications for use of currently available FLT3i in recent EMA and NCCN AML treatment guidelines. Next, we describe the mechanisms of leukemogenesis in this subset and carefully review the main papers reporting molecular NGS and single cell analysis of leukemic resistances recorded in clinical trials and real-life experience. Afterwards we illustrate the more relevant preclinical and clinical studies, investigating new target agents that could change the future perspective of treatment of relapsed/refractory (R/R) FLT3mut AML patients. Table 1 presents the PubMed searches used for study selection.

## 2. FLT3i: Indications, Mechanisms of Resistance, In Vitro and In Vivo Data in Overcoming Resistance

### 2.1. Clinical Trials Analyzing FLT3 Target Therapies in AML

**Figure 2:**
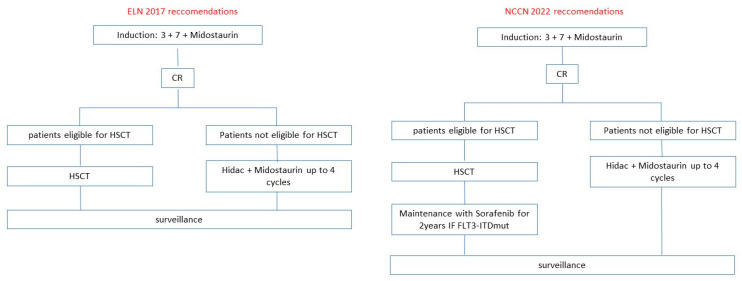
ELN and NCCN recommendations for FLT3mut AML patients fit for intensive treatment.

**Figure 3:**
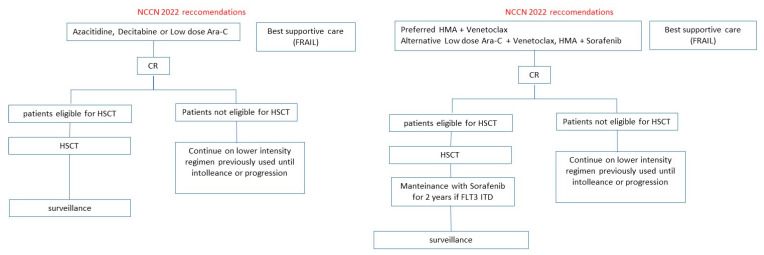
ELN and NCCN recommendations for FLT3mut AML patients unfit for intensive treatment.

Midostaurin and Gilteritinib are AIFA-EMA-FDA approved in naïve FLT3mut AML and relapsed FLT3mut AML, respectively. Other FLT3i such as Quizartinib and Sorafenib were investigated in clinical trials with interesting results but with uncertain evidence of efficacy in terms of survival. The current ELN and NCCN guidelines for FLT3mut AML patients eligible or not to intensive therapy are illustrated in Figure 2 and Figure 3. The results of clinical trials distinguished for FLT3i are illustrated below.

#### 2.1.1. Midostaurin

Midostaurin is a multi-targeted kinase inhibitor active against FLT3 ITD and TKD, platelet-derived growth factor receptor (PDGFR), KIT, SRC, and other RTKs [15].

The randomized phase III RATIFY trial analyzed the efficacy of Midostaurin in combination with the standard backbone therapy including Cytarabine and Daunorubicin (3 + 7) induction and high dose Cytarabine consolidation in patients <60 years with untreated FLT3 (ITD and/or TKD) AML.

The primary endpoint was reached with an HR of 0.78 for OS in Midostaurin arm vs. placebo.

Based on these results, Midostaurin was approved by FDA, EMA and AIFA and now, in combination with intensive chemotherapy, is the new standard of care for the treatment of patients with newly diagnosed FLT3mut AML [16]. In the setting of maintenance, Midostaurin failed to show any benefit in either the RATIFY and RADIUS trials [17].

#### 2.1.2. Gilteritinib

Gilteritinib is a new, multitarget, second-generation type I FLT3i. The phase I–II CHRYSALIS trial showed 41% composite complete remission (CCR) among patients with R/R FLT3mut AML associated with a good safety profile [18]. The randomized phase III ADMIRAL trial evaluated Gilteritinib vs. investigator choice salvage chemotherapy in 371 patients with R/R FLT3mut AML [19]. 

The initial data showed that Gilteritinib decreased the risk of death by 36% and improved both rates of CCR and OS, with an advantage of 3.7 months when compared to salvage chemotherapy [19].

Furthermore, these results were confirmed in a recent study update [20]. Based on these results Gilteritinib monotherapy was approved in US and Europe in patients with R/R FLT3mut AML. 

The MORPHO phase III placebo-controlled trial, evaluating post-HSCT maintenance with Gilteritinib in FLT3mut AML, recently completed enrollment and results are keenly awaited (NCT02997202).

#### 2.1.3. Quizartinib

Quizartinib is a second-generation highly selective type II FLT3i [21]. In a randomized phase IIb trial enrolling R/R FLT3-ITDmut AML patients, Quizartinib in monotherapy showed a 47% response rate [22], giving the rationale for a randomized-phase III trial QuANTUM-R evaluating Quizartinib monotherapy vs. investigator choice salvage chemotherapy in 367 patients with R/R FLT3-ITDmut AML [23].

In this trial, Quizartinib demonstrated a statistically significant OS improvement of 1.5 months in comparison to salvage chemotherapy.

The role of post-HSCT maintenance with Quizartinib (60 mg/d) in FLT3-ITD AML was explored in a phase I study which showed a reduced relapse rate [24].

Given the relatively small OS improvement and the concerns over potential side effects, including cardiac toxicity, Quizartinib was not approved in the US and Europe, but is approved in Japan as a monotherapy in R/R FLT3-ITDmut AML.

#### 2.1.4. Sorafenib

Sorafenib is a first-generation type II multi-kinase inhibitor active against RAS/RAF, c-KIT, vascular endothelial growth factor (VEGF) receptor, PDGFR kinases and FLT3 [25]. 

Due to its broad spectrum of action, Sorafenib was combined with standard chemotherapy in the randomized SORAML trial, where 267 patients ≤60 years with newly diagnosed AML, irrespective of FLT3 status (only 17% had FLT3-ITDmut), received 3 + 7 induction and high-dose Cytarabine consolidation with or without Sorafenib [26].

Patients in the Sorafenib arm had a significantly improved EFS and RFS in comparison to standard chemotherapy with similar OS results, although a recent update suggested a trend for longer OS [27].

In another trial enrolling 27 newly diagnosed FLT3-ITDmut AML patients who were not candidates for intensive chemotherapy, Sorafenib combined with 5-Azacytidine reported a 78% overall response rate (ORR), with a median duration of remission of 14.2 months and an acceptable safety profile [28].

Finally, a randomized placebo-controlled multicenter phase II trial, called the SORMAIN trial, evaluated the role of Sorafenib as a maintenance therapy after HSCT in FLT3-ITDmut AML patients [29]. In this trial, Sorafenib or placebo were administered for 2 years or until relapse or intolerable toxicity. At a median follow-up of 41.8 months, Sorafenib demonstrated higher 2 yr RFS and OS compared to placebo. Patients with negative minimal residual disease (MRD) (MRDneg) pre-HSCT and those with positive MRD (MRDpos) post-HSCT derived the strongest benefit from maintenance with Sorafenib compared to placebo.

Unfortunately, Sorafenib was also associated with higher rate of graft-versus-host disease (GVHD) and skin toxicity compared to the placebo arm. Sorafenib is currently not yet approved in the United States or Europe for the treatment of AML patients.

#### 2.1.5. Maintenance after Allogeneic Transplant

The vast majority of patients affected by FLT3-ITDmut AML, in remission after first-line or salvage chemotherapy, relapse if they do not receive HSCT, which therefore remains the cornerstone of treatment of the disease [30]. Despite this, the recurrence rate after HSCT remains high, up to 75%, representing an unmet medical need. In this regard, maintenance with FLT3i represents a possible resource. It is interesting to understand how much the reduction in recurrence is related to the effect of maintenance on residual leukemic subclones or to the enhancement of graft versus leukemia [31]. Sorafenib has been shown to induce donor CD8 lymphocytes response via activation of the IRF7-IL15 axis in residual FLT3-ITDmut leukemic cells, mediated by suppression of ATF4. This finding has been confirmed in mouse models and in samples of leukemic cells collected from patients responding to Sorafenib [32].

### 2.2. Analysis of Refractory Relapsed Patients after FLT3i Exposure

FLT3-ITD mutations determine the switch from the inactive (so-called “DFG-out”) to the active conformation (DFG-in) of FLT3 receptor. FLT3-TKD (D835) mutations block the receptor in the active conformation (DFG-in), due to the substitution of Asp at position 835 of the activation loop, leading to the opening of the ATP binding and auto-activation of the receptor. Type I FLT3i bind the FLT3 receptor in the DFG-in conformation much more strongly than the DFG-out conformation, either near the activation loop or the ATP binding pocket, and are active against both FLT3ITD and TKDmut AML. Type II FLT3i target the ATP-binding domain of FLT3 receptor, exclusively in the DFG-out conformation, are selectively active against FLT3-ITDmut AML, and resistant to FLT3-TKD mutations. Amino acid changes around the binding site are some of the structural reasons of resistance to type I FLT3i [33,34].

The “gatekeeper” mutation F691L showed universal resistance to all the currently available FLT3i [35]. Sensitivities of FLT3i are summarized in Table 2 [36,37,38].

Here, we describe the scenario of primary and secondary resistance, reported in preclinical study, in real life, and in clinical trials investigating FLT3mut AML treatment.

#### 2.2.1. Mechanisms of Resistance: In Vitro Studies

Traer et al. identified fibroblast growth factor 2 (FGF2) and CXCL12/CXCR4 stromal release as a possible mechanism of resistance to FLT3i [39,40]. The increase in FGF2 might be mediated by Quizartinib in response to drug-induced stromal stress. Increased FGF2 production preceded recurrence and provoked relapse via activation of the RAS-MAPK pathway.

Microenvironment-mediated resistance to Gilteritinib, studied in FLT3mut cell lines, analyzed by integrating WES, unbiased genome-wide clustered regularly interspaced short palindromic repeats (CRISPR-Cas9), metabolomics, proteomics, phosphoproteomics, and small molecule inhibitor screenings, confirmed these data [41]. Early resistance was found to be ligand-dependent, mediated by FGF2 and FL, as well as by alterations of glycerophospholipid metabolism and Aurora kinase B (AURKB) pathway and hyperactivation of the upstream cell cycle regulator of AURKB, CDC7 [42]. In contrast, late resistance was characterized by the emergence of NRAS and MAPK mutations, a finding also confirmed by in vivo experiments [43]. 

Quizartinib resistance, reconstructed in the in vitro model of Dumas et al., was also found to be related to AXL activation via the canonical GAS6 ligand, through soluble STAT5-activating factors, and local hypoxic environment [44].

In addition, the in vitro model of resistance to Midostaurin and Sorafenib showed elevated levels of CCL5, with restoration of response after exposure to the CXCR4 receptor antagonist, Plerixafor [45].

Ras-related C3 botulinum toxin substrate 1 (Rac1) is a protein involved in actin-mediated cytoskeleton remodeling. Midostaurin-resistant FLT3mut cell lines showed overexpression of RAC1, resulting in hyperphosphorylation of N-WASP, inducing actin polymerization, and of the anti-apoptotic protein BCL-2 [46]. In vitro data showed how Midostaurin resistance can be overcome by a combination of Midostaurin, the BCL-2 inhibitor Venetoclax, and the RAC1 inhibitor Eht1864, in FLT3-ITDmut AML cell lines and primary samples.

**Figure 4:**
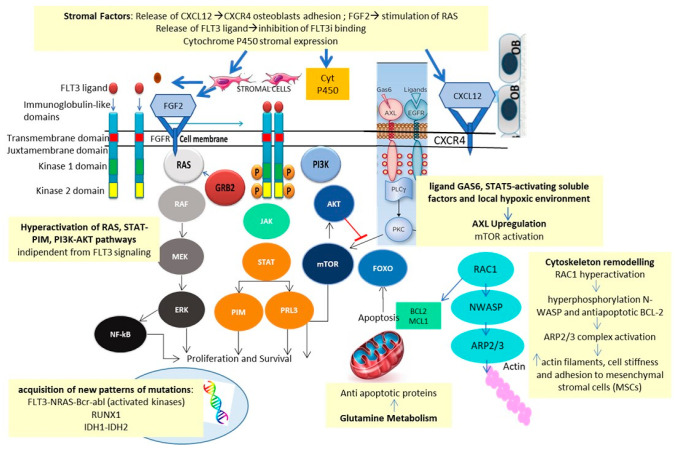
Mechanisms of resistance to FLT3i.

Last but not least, the metabolism of FLT3i is affected by cytochrome P450 3A4 expressed by bone marrow stromal cells and might also be dependent on pharmacological interactions with other drugs, metabolized by cytochrome P450 [47]. Mechanisms of resistance to FLT3i are summarized in Figure 4.

These studies identified MEK, AURKB, CDC7, CCL5, BCL2, RAC1, NRAS and MAPK as possible targets deserving of inhibition for overcoming FLT3i resistance.

#### 2.2.2. Real-Life Experiences

Alotaibi et al. analyzed bone marrow with NGS-based myeloid panel before and after FLT3i-based therapy (Midostaurin, Gilteritinib, Crenolanib, Quizartinib and Sorafenib) in 67 relapsed and 106 refractory patients in a cohort of 946 FLT3mut AML patients treated at MD Anderson [48]. Mechanisms of secondary resistance were identified. Variant allele frequency (VAF) analysis showed that RAS mutations emerged with a higher median level of VAF (32%) in relapsed subset in comparison with responders. These patients had a persistent FLT3 mutation in 74% of cases, but 55% developed emergent mutations, while 26% lost FLT3 mutation, in similar percentage in conventional chemotherapy (CCT) and low intensity (LIT) arms. Epigenetic modifiers (16%), RAS/MAPK (13%), WT1 (7%) and TP53 mutations (7%) emerged after relapse and these latter were more frequent after CCT.

Off-target mutations were more frequent after type I FLT3i, whereas on-target FLT3 mutations occurred in 65% of patients relapsed after type II FLT3i, with a 30% incidence of FLT3-D835, which was reduced when these were associated with CCT compared with LIT.

DNMT3A and IDH2 mutations were more frequent in responders than nonresponders, while RAS mutations with a VAF > 20% were related to refractoriness, particularly to treatment with type I FLT3i.

The authors suggest that this analysis has some bias due to the 1% NGS sensitivity threshold and variability in FLT3i combination therapy administered. Analysis of emerging subclones with droplet digital polymerase chain reaction (PCR) or other ultra-deep sequencing platforms, pre-therapy and at relapse, might help in the future to better understand the mechanisms of relapse.

#### 2.2.3. Sorafenib

F691 and codon D835 mutations were found to be linked to resistance in two clinical trials with Sorafenib [49,50]. In another study, the A848P mutation resulted in secondary resistance to sunitinib and Sorafenib, but not to Midostaurin [51]. Overexpression of the kinases PIM-2 and AXL were also likely to constitutively activate STAT5, causing resistance to Sorafenib.

#### 2.2.4. Midostaurin

N676K mutation, inducing a single amino acid substitution within the FLT3 kinase domain, was the first to be identified as resistance-leading mutation in a patient with R/R AML treated with Midostaurin [52].

**Figure 5:**
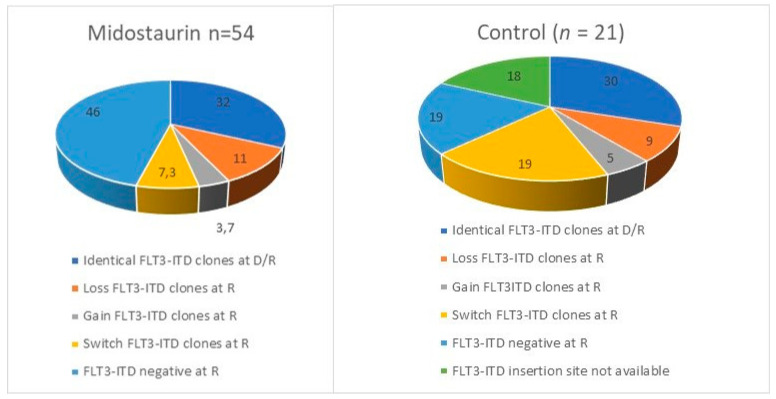
Distribution of FLT3 mutations at relapse or progression in RATIFY trial [34]. D/R: diagnosis/relapse or progression; R: relapse or progression.

The Midostaurin registrative phase III trial RATIFY showed a 59% CR rate in the experimental arm with 40% resistance and 40% of relapse. Genescan-based testing for FLT3-ITD and whole exome sequencing (WES) were performed at diagnosis and relapse or resistance in a selection of 54 patients receiving Midostaurin and chemotherapy and 21 treated with chemotherapy alone, enrolled in RATIFY or the German–Austrian Acute Myeloid Leukemia Study Group 16-10 trial [53]. Relapsed and refractory patients lost *FLT3*-ITD clone in 46% of cases treated with Midostaurin compared to in 19% in cases who did not receive it. Switched or gained FLT3ITD clones emerged in the Midostaurin group in 11% of relapsed patients vs. 0% of refractory patients, suggesting that the acquisition of new FLT3 mutations is associated with the duration of Midostaurin exposure. FLT3 mutations were stable at relapse in 32% of patients treated with Midostaurin vs. 48% of naïve patients. In patients with FLT3-ITD persistence, selection of resistant ITD clones was found in 11% as a potential driver of disease. Figure 5 shows how the repertoire of FLT3 mutations changed at relapse or progression in the Midostaurin and control arms of the RATIFY study.

At the onset of resistance or disease progression, the Midostaurin group presented fewer on target than off target mutations.

The pathway enrichment analysis detected activation of RAS and MAPK in resistant patients, losing FLT3 mutation at relapse. Mutations of WT1 (*n* = 3), RUNX1 (*n* = 3), RAS (*n* = 4), IDH1 (*n* = 2), chromatin/splicing related genes (ASXL1, U2AF1, ZBTB7A and SF3B1; *n* = 4) were identified in resistant patients at WES analysis, while only two relapsed patients had the N676 mutation, with low VAF (<5%).

In contrast, refractory patients had persistence of mutations present at diagnosis, with activation of genes related to cell cycle regulation (CCND3, SMC1A, RAD21, CDKN1C). NPM1 expression was found to be doubled in relapsed compared to refractory patients (43% vs. 21%), while WT1 mutations were equally distributed in the two groups.

#### 2.2.5. Quizartinib

Secondary resistance to Quizartinib is influenced by ineffectiveness against D835 and the gatekeeper residue of kinase mutations, F691L, with consequent selection of related clones [54]. Single cell analysis identified the emergence of several subclones with ITD and D835V, Y, F mutations or with different TKD mutations alone. These results suggest that resistance may be polyclonal, and that single cell analysis is the best method for understanding the mechanisms of relapse [55].

#### 2.2.6. Gilteritinib

The ADMIRAL trial’s authors recently published interesting results concerning patients with FLT3mut AML relapsing after Gilteritinib.

Acquisition of new mutations occurred in 40 patients: 18 involved RAS/MAPK pathway, 6 FLT3 (5 F691L), 3 WT1 (1 with F691L), 1 IDH1, and 1 GATA2; 13 patients (32.5%) had no new mutations. The acquisitions of RAS/MAPK pathway gene mutations and FLT3 F691L gate keeper mutations at relapse were mutually exclusive [43]. Not transplanted patients gained RAS/MAPK and FLT3 F691L mutations at relapse, but these formers did not correlate with refractoriness.

The correlation between frequency of emergent FLT3 F691L gatekeeper mutations at relapse and dose of Gilteritinib is unclear. In the Admiral trial, patients who received 120-mg/day Gilteritinib had a similar incidence of FLT3 F691L incidence compared to that observed in relapsed patients who received 20 to 200 mg/day Gilteritinib, while none of the patients receiving >200 mg/day Gilteritinib acquired the mutation at relapse. Nevertheless, patients receiving 120 mg/day had better OS compared to other patients [56].

Another study showed a correlation between Gilteritinib dose and resistance in 22 FLT3mut AML patients analyzed at relapse by NGS and single cell analysis, identifying in those receiving doses below 200 mg, a more likely development of RAS or FLT3 F691L mutations [57].

#### 2.2.7. Crenolanib

Crenolanib is a second-generation type I FLT3i active against FLT3/PDGFR at concentrations lower than those reported as safe in humans [58]. Zhang et al. performed WES of samples from R/R FLT3 pos AML patients before and after Crenolanib administered in a phase II study (NCT 01522469, NCT 01657682) [59]. Patients resistant to Crenolanib treatment rarely showed FLT3TKD mutations, except for F691L mutations. One resistant patient showed a novel extracellular FLT3 mutation, K429E, with elevated VAF. Two different pathways of clonal evolution were observed: a linear one with acquisition of TET2 and IDH1 mutations in clones with persistent FLT3 mutations and a branching evolution with acquisition of NRAS and IDH2 mutations in FLT3-independent subclones. RAS was more frequently mutated in patients pretreated with FLT3i, who were less responsive to Crenolanib than naïve patients. Resistant patients also acquired mutations in epigenetic regulators, transcription, and cohesion factors. Drug combinations in experimental models restored sensitivity to Crenolanib, and clinical trials therefore used it in combination with cytotoxic chemotherapy, both in first line and relapse. Overall response rate of 36% was observed in 13 patients with R/R FLT3mut AML after high doses of Cytarabine and Idarubicin plus Crenolanib [60]. In first line, the combination of Crenolanib with standard “7+3” induction and consolidation with high-dose Cytarabine resulted in 96% CR+CRi, with 88% CR [61] with a median follow-up duration of 14 months, suggesting durable responses with this combination.

### 2.3. Overcoming Resistance

Evaluation of new FLT3i, the combination of several target agents, and the use of multi-target agents represent possible future approaches to overcoming AML FLT3 resistance. Here, we report the most representative in vitro studies and the enrolling and not yet enrolling clinical trials available.

#### 2.3.1. New Compounds

Knowledge of the FLT3 receptor sites susceptible to the most relevant mutations, conferring resistance to I and II generation FLT3i, guided the construction of new inhibitors capable of bypassing these resistances. In vitro studies using cell lines and xenograft models have validated their efficacy. Below, we have selected several studies [62,63,64,65,66,67,68,69,70,71,72] that have identified novel FLT3i with interesting efficacy and selectivity data, probably leading actors in future clinical trials. Table 3 summarizes the preclinical studies analyzing new compounds with their sensitivities and resistances.

#### 2.3.2. Combinations of Different Target Agents

FLT3i showed several mechanisms of resistance and single cell and NGS analyses showed the presence of multiple complexity in the leukemic escape suggesting the emergence of multiclonal or oligoclonal resistant AML cells at relapse. Scientists are all converging on attempting to bypass potential FLT3i failures by using the association of different FLT3i or the combination of FLT3i with chemotherapy and target or multitarget agents. The principal pathways explored in preclinical studies are summarized in Table 4.

##### FLT3 Inhibitors

Bregante et al. identified 2 out of 18 compounds active against FLT3-ITD AML, WS6 and Ispinesib, and combined them with two approved drugs, Ponatinib and Cabozantinib, in in vitro models (AML cell lines and samples) [73]. WS6 had a similar mechanism and potency to Ponatinib and Cabozantinib. Interestingly, Ispinesib and Cabozantinib inhibited AXL, known as a possible driver of FLT3-ITD AML drug resistance. They concluded that in vitro synergy of WS6, Ispinesib and Cabozantinib or Ponatinib in FLT3-ITDmut AML could be the rational background of future clinical trials with combinations of these drugs.

##### FLT3i and BET Inhibitors

BET inhibitors play an important role in suppressing leukemogenesis through inhibition of leukemic pro-survival factors such as MYC and BCL2 but insufficient single-agent clinical potential and low specificity and hematological tolerance related to activity in normal bone marrow cells are reasons of concern. Lee et al. showed that the novel 4-azaindole derivative PLX51107 has BET-inhibitory activity in vitro (MYC plasma inhibitory activity assay in OCI-AML3 cells for BET inhibition and FLT3 plasma inhibitory activity assay in MOLM-14 cells) and in vivo (MV4-11 mouse xenograft model) [74].

Tumor growth was significantly inhibited in mice treated with **Quizartinib-PLX51107** compared to mice treated with 5 mg/kg Quizartinib alone. PLX51107 appears to be the ideal BET inhibitor because of its short plasma half-life, resulting in high specificity against leukemic cells, compared with normal bone marrow precursors, permitting a safe combination with continuous FLT3i exposure. The association of Quizartinib-PLX51107 could be further investigated in future clinical trials.

##### Multiple Tyrosine Kinase Inhibitors

Quizartinib efficacy is hampered by bone marrow stromal niche through STAT3 and STAT5 activation. Patel et al. attempted to reproduce this leukemic protective environment in an in vitro model of FLT3-ITD+ AML cells cultured in conditioned medium, obtained from bone marrow stromal cells cultures [75]. They concluded that the synergy between Dasatinib and Quizartinib was STAT5 independent, as it was not abolished by the knockdown of STAT5 mediated by Doxycycline. An Israeli study identified FLT3/ITD and PTPN11 mutations as predictors of Dasatinib sensitivity, whereas TP53 mutation was found to be associated with Dasatinib resistance at CRISPR-Cas9 analysis. The authors also found that Dasatinib had an antileukemic effect on leukemic stem cells (LSCs) of FLT3-ITD AML samples injected into NSG-SGM3 mice. Dasatinib might therefore be combined with FLT3i in FLT3/ITD, PTPN11-mutated AML [76].

##### FLT3 + AKT-MTOR/HSP-MEK Inhibitors

Fleischmann et al. studied the two distinct phosphoproteome patterns in human FLT3 mut AML (MOLM13) and murine AML cell lines (Ba/F3), depending on the localization of FLT3ITD [77]. Pretreatment with glycosilation inhibitors Tunicamycin and 2-deoxy-D-glucose resulted in an endoplasmic reticulum localization of FLT3ITD protein, with a consequent upregulation of chaperone proteins HSP90beta1 and GRP94 and activation of ERK. Incubation of cell lines with the histone deacetylase inhibitor Valproic Acid increased surface expression of FLT3ITD through glycosilation and upregulation of the 150 KD FLT3ITD isoform, which downstream decreased ubiquitin protein ligase E3 NEDD4 and increased PKCdelta, with consequent phosphorylation and activation of AKT in the PI3K-AKT-mTOR pathway.

In conclusion, two different patterns of localization of FLT3 are associated with two specific phosphoproteome and chemosensitivity settings:

→ the surface pattern responds better to AKT-mTOR inhibitors Rapamycin
→ the endotelial reticulum pattern might benefit from chaperones (HSP90) and MEK inhibitors. The authors also advocated a synergistic interaction between Valproic Acid and MEK inhibitors.

Huang et al. analyzed in vitro activity of PI3K inhibitor, LY294002, in Sorafenib resistant FLT3 mut AML cell lines (BaF3-ITD-R) [78]. Loss of FLT3 and persistent activation of the downstream PIK/AKT signaling enhances glycolytic activity, ATP production and leukemic cell survival, making PIK/AKT a possible target for leukemic relapse. Other PI3K and AKT inhibitors did not have the same efficacy, suggesting a multiple and complex mechanism of action of LY294002. 

##### FLT3 and JAK2 Inhibitors

Momelotinib is a JAK2 inhibitor also active on FLT3 that has recently shown efficacy in FLT3i-resistant cell lines such as Gilteritinib and Quizartinib, expressing mutations (FLT3 D835, D839 and Y842) [79]. A recent study showed the emergence of JAK mutations in cell lines resistant to Midostaurin and Sorafenib, and sensitive to dual FLT3/JAK inhibition, confirming the rationale of combining a dual FLT3/JAK inhibitor with a FLT3i [80].

##### FLT3 and Histone Deacetylase Inhibitor

A recent study showed that FLT3i can determine activation of histone deacetylase 8 (HDAC8) via FOXO1 and FOXO3, blocking p53 and themselves providing an escape from apoptosis, and thus a mechanism of resistance [81]. Inhibition of HDAC8 by compound 22d was shown to significantly reduce the engraftment of primary FLT3-ITDmut AML cells in Quizartinib-treated mice, providing the rationale for the combination of the two drugs.

##### FLT3 and MDM2 Inhibitors

MDM2 is an oncogenic protein inhibiting normal p53 function. Therefore, MDM2 inhibitors retain their activity only in TP53 wild type AML, because they are unable to interact with deleted or absent p53. Seipel et al. analyzed the in vitro efficacy of MDM2 inhibitor **NVP-HDM201 in combination with Midostaurin**, demonstrating significantly increased susceptibility to FLT3i in NPM1 and TP53 wild type FLT3mut AML cells with high allelic ratio. The combination NVP-HDM201 and Midostaurin was as effective as chemotherapy + Midostaurin in FLT3-ITD positive TP53 wild type cells, suggesting a possible role in future clinical trials [82].

##### FLT3 and AXL Inhibitors

Among the AXL inhibitors [83,84], the small molecule BGB324 (R428) was shown to increase the in vitro sensitivity of AML cells to Doxorubicin and Cytarabine [85], and is currently under evaluation in a multicenter phase Ib/II clinical trial alone or in combination with Cytarabine/Decitabine in high-risk myelodysplastic syndromes and R/R leukemia (NCT02488408). DAXL-88 antibody [86], its monomethyl auristatin E (MMAE) conjugate DAXL-88-MMAE [88], and R428 were assayed in vitro against drug-resistant AML cell lines and FLT3-ITD-TKD blastic AML cells.

Liu et al. selected drug-sensitive and drug-resistant human AML cell lines and FLT3-mut AML blast cells with high AXL antigen expression to analyze the cytotoxic effects of DAXL-88, DAXL-88-MMAE and R428 [87]. Drug-resistant AML cell lines and FLT3-ITD-TKD AML blast cells showed an upregulated AXL antigen. AXL-targeted agents inhibited the growth of FLT3mut AML cell lines and FLT3-ITDmut AML primary samples in a dose-dependent manner, and synergistically inhibited proliferation and induced apoptosis of MV4-11/AC220 and FLT3i-resistant AML blast cells when combined with Quizartinib. DAXL-88 and DAXL-88-MMAE were found to be able to inhibit AXL, FLT3 and their downstream signaling pathways. The authors suggested, as the final mechanism of action, a steric hindrance block of the binding of AXL to FLT3 in FLT3mut AML cells with the inhibition of AXL heterodimerization, and phosphorylation of AXL, FLT3 and their downstream molecules AKT and ERK.

##### FLT3 and Menin Inhibitors

Based on the previous evidence of the downregulation of MEIS1 and its transcriptional target gene FLT3 by inhibitors of the menin-MLL complex, MI-503 was tested with FLT3i Ponatinib and Gilteritinib, demonstrating synergism in suppressing FLT3 and downstream genes [89]. This synergistic inhibition was confirmed in human and mouse models of FLT3mut leukemias with NPM1 (MI-503, VTP-50469 and Ponatinib/Gilteritinib) and MLL-r (MI-503, VTP-50469 and Quizartinib) with increased antileukemic efficacy determined by the combination of the inhibitors compared to single drug treatment. Combined inhibition of menin-MLL and FLT3 represents a promising new therapeutic strategy for patients with FLT3mut leukemia with NPM1mut or MLL mutation.

#### 2.3.3. Rotating FLT3 Inhibitors

Yang et al. studied the effect of Quizartinib and Pexidartinib rotation in AML cell lines (MOLM-14 and MV4-11) by analyzing the onset of resistance using computational studies [90]. They observed that the efficacy of both inhibitors quickly reverted to resistance with no benefit from any rotation scheme. F691L is the most common mutation acquired after Quizartinib, and it was not prevented from rotation of the two target agents.

#### 2.3.4. Multitarget Agents

##### Multiple FLT3 AXL MET VEGFR KIT Inhibitors

Cabozantinib is an oral multitarget inhibitor of FLT3, AXL, MET, VEGFR, and KIT showing a potent inhibition of FLT3-ITD mut cell lines with D835 resistant mutations [91]. It is already approved for the treatment of patients with progressive metastatic medullary thyroid cancer, with hepatocellular carcinoma after Sorafenib and adults with advanced renal cell carcinoma who are treatment naïve with intermediate or poor risk, or who have received prior VEGFR targeted therapy [92]. The preclinical results in AML and clinical benefit, achieved in the treatment of other cancers, suggest a possible role of Cabozantinib in future clinical trials enrolling R/R FLT3 mut AML.

##### Dual FLT3 and JAK2 Inhibitors

Compound 14J, derived from the pharmacophore assemblage of momelotinib and tandutinib, demonstrated enhanced in vitro inhibition of JAK2 and FLT3 in AML cell lines [93], enough to suggest its suitability for its use in future clinical trials.

##### Dual FLT3 and Aurora Inhibitor

CCT241736 is an oral dual inhibitor of FLT3 and Aurora kinase that also showed efficacy in leukemic cell lines and xenograft models of FLT3-ITDmut and or FLT3-TKDmut tumors resistant to Quizartinib and Sorafenib [54,94]. The drug has significant anti-FLT3 and Aurora kinase activity and selectivity, making it a good candidate for use in clinical trials in FLT3-ITD and TKDmut AML resistant to previous treatments.

##### Dual FLT3 and AMPKα Inhibitor

Wu-5 is a novel inhibitor of USP10 that induces degradation of FLT3-mutated protein and downregulates compound C AMPKα, which was shown to effectively inhibit proliferation of FLT3mut cell lines such as MV4-11, Molm13 and MV4-11R [95].

Wu-5 and Crenolanib showed synergism in the inhibition of FLT3 and AMPKα in FLT3-ITDmut cells, while metformin hampers the efficacy of Crenolanib due to the activation of AMPKα, confirming the interactions between Crenolanib and AMPKα activity.

##### Dual FLT3 and Tubulin Inhibitor

Wang P. et al. identified and analyzed the in vitro efficacy of a dual oral FLT3 and tubulin inhibitor KX2-391, with very interesting effects on resistant FLT3mut AML cell lines (D835 and F691L). It also effectively reduced leukemic growth of FLT3-ITD-F691L, FLT3-ITD and FLT3-ITD-D835Y mut AML cells in a xenograft leukemia model [96].

##### Dual FLT3/Hedgehog Inhibitors

Xu et al. identified a new FLT3/Hedgehog inhibitor, called Triptonide, with interesting abilities to specifically inhibit FLT3-ITDmut AML cells, sparing normal cells. The small molecule induced cell cycle arrest at G0/G1 and apoptosis of MOLM-13 cell line in in vitro and in vivo murine xenograft model [97]. Proteomic and genomic studies demonstrated the inhibition of the oncogenic protein GLI2, with c-Myc decreasing and p53 increasing. GLI2 is the key effector of Hedgehog signaling with an important role of c-Myc and p53 regulation and Triptonide could be an interesting compound to explore in basic research and translational studies.

#### 2.3.5. Immunotherapy

CAR-T are the most exciting immunological bullets currently available for R/R acute lymphoblastic lymphoma and R/R Non-Hodgkin Lymphoma. Li K. et al. constructed FLT3scFv/NKG2D-bispecific-CAR T cells, including a new CAR construct comprising the extracellular region of the human NKG2D receptor and the FLT3scFv, that showed cytotoxicity against FLT3mut AML cells and significantly prolonged the survival of the MOLM-13 cells engrafted mice. The bispecific CAR T cells eliminated both primary FLT3mut and FLT3wt AML blasts, although the treatment effect on the FLT3mut AML blasts was more profound. The tumor-killing efficacy of CAR T cells improved dramatically when they were administered in combination with Gilteritinib both in vitro and in vivo [98].

#### 2.3.6. Phase I Studies

Here, we report results of recent phase I trials with new FLT3i or target agents.

##### Pacritinib + 3+7/Decitabine

A phase I study explored twice-daily administration of 100 mg vs. 200 mg of the oral JAK2/FLT3i Pacritinib in combination with 3+7 in five patients with first-line FLT3 AML (cohort A) or in combination with 10-day cycles of Decitabine in eight patients with R/R FLT3 AML (cohort B). Pacritinib administration schedule was from day 1 to day 4 and from day 8 to day 21 in cohort A (with 3+7 infusion from day 5 to day 11), and from day 1 to day 21 in cohort B (with 20 mg/sm Decitabine infusion from day 5 to day 14). There was one early death, two CRs, and two stable disease responses in cohort A, and one morphological leukemia-free state and five stable disease in cohort B, with two cases of dose-limiting toxicity at the 100 mg dose, due to hemolytic anemia and grade 3 QTc prolongation, respectively. The median OS was 292 days, and two CR patients in cohort A were safely transplanted. A 35% reduction in blasts was observed in two patients after pretreatment with Pacritinib in cohort A, suggesting interesting activity even in monotherapy. The study’s low sample size and preliminary results warrant further investigation [99].

##### Pexidartinib

The activity of the oral FLT3i Pexidartinib, with interesting in vitro efficacy against the conferring resistance mutation F691L, was explored in a phase I trial with a dose escalation phase at daily doses ranging from 800 to 5000 mg in 34 patients, without dose limiting toxicities, followed by a dose expansion phase of 3000 mg daily dose in 56 R/R FLT3 AML patients [100]. Diarrhea (50%), fatigue (47%), and nausea (46%) were the most frequent adverse events, but febrile neutropenia (12%), sepsis (6%) and increased aspartate aminotransferase (6%) were the most frequent events resulting in dose changes. Grade 5 adverse events were seen in 13% of patients, not related to treatment in all but one case, due to differentiation syndrome. The authors observed 21% ORR, with an overall CCR rate of 11% and 6.7% transplant rate, 25% of CR in F691Lmut patients. The median OS was 112 days with 265 days in responder in the dose expansion arm, without differences between FLT3i pretreated and naïve patients.

##### Sorafenib and Plerixafor + G-CSF

The strategy of increasing leukemic killing through leukemic FLT3mut cells mobilization was investigated in a phase I study combining oral FLT3i Sorafenib at 400, 600 and 800 mg twice daily, with G-CSF and plerixafor administered every other day for seven doses starting on day 1. The trial enrolled 28 patients with R/R FLT3-ITDmut AML, 36% after HSCT and 39% after failing a previous FLT3i treatment, including one patient refractory to sorafenib. Extrahematological ≥ grade 3 treatment-related events were reported in 20 patients mainly due to skin rash, arrhythmia, elevation of liver enzymes, bone pain, and, less frequently, transient renal failure, pleural effusions and pericardial effusions. The CCR rate was 37%, with a median duration of response of 5.3 months. Two patients (7%) achieved negativity of FLT3, 1 is still in remission at 56 months and 1 relapsed after 16 months after acquisition of D835 mutation. The mobilization of leukemic FLT3mut cells represents an intriguing mechanism of resistance escape deserving further investigation [101].

##### Sorafenib and Omacetaxine Mepesuccinate

Protein synthesis could be a target for overcoming FLT3i resistance. Omacetaxine mepesuccinate OME, able to inhibit t-RNA binding to ribosomes and t-RNA transcription, showed in vitro synergy with FLT3 [88]. Treatment was feasible even in the elderly and surprisingly effective in combination with Sorafenib (SOME), achieving 72% CR/Cri in R/R FLT3-ITDmut AML, 33% transplant rate, 43.6 weeks median overall survival and 22.4 weeks leukemia-free survival among responders [102]. Combination with Quizartinib (QUIZOM) is under investigation in a phase II trial (NCT03135054), with preliminary results showing an enhanced efficacy [103].

#### 2.3.7. Phase II Trials

As previously shown, BCL-2 overexpression is one of the mechanisms of resistance to FLT3i and a potential target of inhibition for overcoming resistance.

Konopleva et al. investigated the triplet Venetoclax-10 days Decitabine-FLT3i in 12 young R/R FLT3mut AML and in 13 patients, aged >60 years, in frontline FLT3mut AML in a phase II trial (NCT03404193) [104]. The principal dilemma was the definition of the median dose and duration of FLT3i during cycle 1 and subsequent cycles. Sorafenib was administered 400 mg bis in die (BID) for 15 days, Midostaurin 50 mg BID for 15 days and Gilteritinib 120 mg for 14 days. For subsequent cycles, Sorafenib was administered 400 mg BID for 14 days, Midostaurin 50 mg BID continuously and Gilteritinib 120 mg daily continuously. Frontline patients achieved 92% CCR rate with 56% and 91% MRD negativity by FCM and PCR/NGS, respectively. In R/R AML the CRc rate was 62%, with 63% and 100% MRD negativity rate according to FCM and PCR/NGS analyses, respectively. Rate and deepness of response were maintained even in the patients pretreated with FLT3i. Early mortality was negligible, with interesting 2 yr OS of 80% in frontline and median OS of 6.8 months in second line patients. R/R patients had a very poor prognosis including 70% of cases relapsing after FLT3i and 30% relapsing after HSCT.

Doublet regimens had similar results, but with shorter follow-up and in less unfavorable settings. Venetoclax associated with Gilteritinib showed CRc of 85% in a phase IB trial enrolling R/R FLT3mut AML [105]. Moreover, non-Venetoclax-based doublet regimens incorporating Sorafenib, Quizartinib, or Gilteritinib with LIT showed comparable results both in frontline [28,106,107] and second-line settings [108].

**Figure 6:**
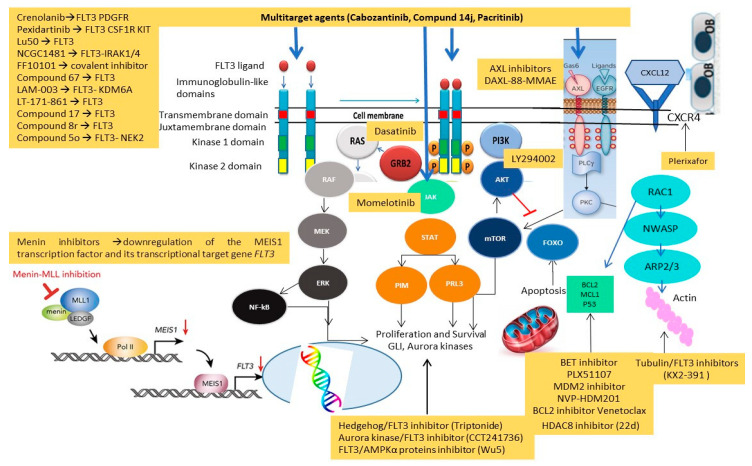
New target agents investigated in preclinical and phase I–II trials and their mechanisms of action.

Despite similar results, authors suggested a potential advantage of triplet vs. doublet FLT3i and LIT combinations, due to deeper responses and prevention of secondary resistance. To reduce the hematological toxicity of the triplet, a bone marrow aspirate at day 14 is recommended to assess blast clearance or aplasia requiring Venetoclax discontinuation. Figure 6 illustrates new target agents investigated in preclinical and phase I–II clinical trials, along with their mechanisms of action.

#### 2.3.8. Future Directions and Ongoing Clinical Trials

Despite the high FLT3 inhibitory efficacy reported in in vitro studies, second-generation FLT3i, Gilteritinib, Quizartinib and Crenolanib showed primary and secondary resistance in FLT3mut AML treatment. The presence of NRAS mutations at baseline or at relapse after FLT3i therapy, and the identification of F691L mutations, represent the most frequent events in those patients requiring new combination therapies.

Table 5 and Table 6 summarize ongoing clinical trials including FLT3i and new target agents combined with chemotherapy or hypomethylating agents (HMA) in FLT3mut AML patients.

The antiBCL2 agent Venetoclax, one of the most interesting approved drugs in recent AML treatment scenarios [109], was associated to Quizartinib and Gilteritinib in both first and second-line treatment of FLT3mut AML. Konopleva et al. showed similar results in FLT3mut AML patients compared to FLT3 wild type AML in a post hoc analysis of the VIALE-A (NCT02993523) and phase Ib trial (NCT02203773) confirming efficacy in this unfavorable setting [110]. The increase in apoptosis is the rational of the association of Venetoclax with FLT3i and HMA or low dose Cytarabine. To date, the optimal schedules of Venetoclax associated to targeted agents, the use of concomitant or sequential administration of these agents, the ideal timing of bone marrow evaluation, and the indications for growth factor support have to be clarified. Hopefully, triplet therapies will improve efficacy, while maintaining an acceptable safety profile with early mortality rates <5–10%. The other new entry in the AML treatment repertoire, CPX-351, is currently available in association with Gilteritinib in a phase I and III study in the R/R and frontline setting respectively.

Randomized phase III studies of CCT in combination with Midostaurin versus Gilteritinib (NCT03836209) and with Midostaurin versus Crenolanib (NCT03258931) are currently ongoing to establish which FLT3i should be used in frontline. Furthermore, phase III study of Gilteritinib versus placebo and phase II Crenolanib trials are ongoing and may help to address the benefit of FLT3 inhibition more definitely as maintenance therapy after HSCT in FLT3mut AML (BMT CTN 1506; ClinicalTrials.gov identifier: NCT02997202, NCT02400255).

The complexity of the mechanisms of resistance to FLT3i just described is highlighted by the table of clinical trials with new target inhibitors (Table 5). Multitarget inhibitors Dubermatinib, Nintendanib, CG-806 and NMS-03592088, BTK inhibitor TL-895, MDM2inhibitor KRT2, oral SYK inhibitor Lanraplenib, checkpoint kinase 2 inhibitor PHI-101, and IRAK4 inhibitor CA-4948 are among the new drugs being evaluated in clinical trials currently available.

Recent advances in immunotherapy have also determined the upgrade of the FLT3mut AML treatment armamentarium by a FLT3 CART and antiCD3/FLT3 bispecific dual-affinity Re-targeting antibody (DART).

## 3. FLT3i: The Past, the Present, the Future

### 3.1. FLT3i: The Past

In vitro and in vivo studies confirmed the polyclonal nature of resistance to FLT3i, which is due to microenvironment factors, alterations of glutamine metabolism, cytoskeleton remodeling and hyperactivation of several downstream pathways of FLT3 receptor. Phase III trials and post hoc analyses of real-life experiences have shown correlations between relapses and mutations of RAS and F691L, and between refractoriness and persistence of FLT3 mutations and acquisition of mutations of IDH, RAS and genes controlling cell cycle and splicing of RNA. RAS mutations were predictive of refractoriness after Crenolanib but they were not after Gilteritinib, while D835 mutations were acquired at relapse after treatment with type II FLT3i.

### 3.2. FLT3i: The Present

Preclinical studies have investigated several new compounds able to overcome the classical mutations conferring resistance to FLT3i, such as F691L and FLT3 D835. The combinations of FLT3i and other target drugs or multitarget agents inhibiting BCL2, MYC, PTPN11, MEK, MDM2, HDA8, Aurora kinases, JAK2, JAK1 and AXL were tested in in vitro and xenograft models. WS6 showed synergy with Ispinesib and Cabozantinib, as Dasatinib did with Quizartinib, especially in AML with PTPN11 mutations. Valproic Acid, MEK and HSP90 inhibitors showed synergy when FLT3 was localized in the endothelial reticulum, while AKT-mTOR inhibitors, such as Rapamycin, are active when FLT3 is situated on the cell surface. The JAK2 inhibitor Momelotenib and the MDM2 inhibitor NVP-HDM201 confirmed their activity, respectively, in JAK2-mutated AML and NPM1 and TP53 wild type settings, while Menin inhibitors were active in MLL and NPM1 comutated FLT3AML. AXL inhibitors target AKT/ERK downstream signaling with interesting results.

Among the multitarget agents, we selected Cabozantinib (multitarget inhibitor), Compound 14J (JAK2i/FLT3i), Wu-5 (AMPKa protein inhibitor/FLT3i), CCT241736 (Aurora kinase inhibitor/FLT3i), KX2-391 (tubulin inhibitor/FLT3i) and Triptonide (Hedgehog inhibitor/FLT3i) as the most interesting drugs, active in in vitro and xenograft models of R/R FLT3mut AML. The bispecific CAR T cells FLT3scFv/NKG2D showed synergy with Gilteritinib, providing a new option of cell therapy, hitherto unexplored in this setting. Clinical phase I trials investigated Pacritinib (JAK2/FLT3i) with HMA and chemotherapy, Pexidartinib, the combinations of Sorafenib with Plerixafor/G-CSF and Omacetaxine mepesuccinate, identifying this latter combination as the one providing the best ORR rate of 72%. Phase II trials, investigating triplet combinations of Venetoclax, HMA and FLT3i showed a low early mortality and very high CR rates in first and second line, burdened by high relapse rates, even after HSCT, in R/R setting. The day +14 bone marrow blast count helped to modulate hematological toxicity by reducing the duration of Venetoclax treatment. The depth and duration of response, especially in newly diagnosed patients, make this approach an attractive option for future phase III trial.

### 3.3. FLT3i: The Future

New FLT3i have shown interesting activity against mutations which confer resistance, such as Compounds 17, 8r, 5o [70,71,72], but current molecular studies, investigating resistance in FLT3mut AML patients, relapsing after treatment, suggest that the persistence or the selection of one or more subclones, are the natural evolution of target inhibition, and that combination with other agents, with different mechanisms of action, is necessary to overcome resistance. The R/R setting still remains an unmet medical need, because of the high relapse rate observed even after HSCT. In the future, new combinations of FLT3i with inhibitors of JAK2, MEK2, HDAC, Menin, AXL and MDM2 or with multitarget agents here reported [73,74,76,77,78,79,81,82,83,87,91,93,94,95,97] and immunotherapies, such as checkpoint inhibitors, vaccines, and adoptive T-cell therapies, may decrease the burden of residual disease and reduce the incidence of relapse and refractoriness.

We are waiting for results of ongoing clinical trials investigating combinations of Venetoclax and or HMA, CPX-351 and chemotherapy with FLT3i in newly diagnosed and R/R patients. These data might therefore change the paradigm of the cure of the disease, which still represents an unmet medical need, especially in a second-line setting.

Anti-FLT3/CD3 DART and anti-FLT3 CAR-T are some of the current specific ‘immune magic bullets’ available in ongoing clinical trials in the R/R FLT3mut AML treatment scenario. However, future trials could also select and investigate well-tolerated possible specific antibody-based immunotherapies, with the aim of eradicating LSCs or pre-emptively treating molecular relapse. CD123 is frequently expressed in AML and CD99 was recently found to be specifically expressed by FLT3mut LSCs [111]. Bispecific DART antibody-based molecule to CD3ε and CD123, Flotetuzumab, has already shown interesting results, with a 30% CR in R/R CD123+ AML setting, while anti CD99 antibody has not yet been investigated [112]. Nevertheless, the nanoworms α-CD99-A192, a fusion protein composed of a single-chain variable fragment antibody (anti-CD99 scFv), conjugated with a high-molecular-weight elastin-like polypeptide (ELP) A192, demonstrated excellent in vitro and in vivo anti-leukemic effects in AML cell lines, primary blasts, and xenograft mouse model [113].

Post-HSCT maintenance administration is really an intriguing topic because of the frequency and poor prognosis of post-HSCT relapse. However, due to the uncertainty of Sorafenib safety data, we are still waiting for a better FLT3i in this context [29]. This has inspired a multitude of other studies investigating the role of other FLT3i in post-HSCT maintenance, the results of which have not yet been published.

The presence of comutations is another important factor to consider when choosing induction therapy. FLT3 and IDH mutations can be co-expressed at diagnosis, and Shoukier et al., on the basis of a real-life experience, suggested that the value of VAF may guide the choice of the best target therapy between FLT3i and IDH inhibitor [114]. As a matter of fact, the efficacy and safety of a combination of two target drugs has not yet been investigated.

NPM1 and FLT3 comutations represent another intriguing subset with more favorable outcome than NPM1 wild type FLT3mut AML. Menin inhibitors showed a potential activity in NPM1mut AML, related to MLL1 and MLL1-fusion protein inhibition. Menin inhibitors also inactivate MEIS1 transcription factor with the particularly interesting effect of downregulating its transcriptional target gene FLT3, suggesting a possible synergy with FLT3i, especially in NPM1mut-FLT3mut AML, and also in MLL-FLT3mut AML [115]. Researchers have recently demonstrated that the inhibitory effect of menin inhibitors on BCL2 protein is synergistic with that of Venetoclax in NPM1mut-FLT3mut AML [116].

Inhibition of the antiapoptotic protein BCL2 is another important available therapeutic option for overcoming resistance. Double or triplet regimens can increase the depth of response and avoid persistence or the appearance of leukemic subclones. Administration of oral target agents and hypomethylating agents can spare chemotherapy and improve quality of life and the psychological impact of the disease by reducing extrahematological toxicity and increasing time spent out of the hospital, especially in patients who are not transplant candidates. Careful monitoring of quality-of-life-adjusted costs is necessary in future clinical trials, as it is already known that new regimens come with the burden of high costs [117].

## 4. Conclusions

We have seen how the acquisitions of secondary mutations can cause FLT3AML relapse through a linear evolution if they occur in the original FLT3mut clone, or through a branching evolution if they arise in a clone different from the original FLT3mut leukemic clone. Clinical trials and real-world experience using FLT3i have reported high rates of acquisition of RAS and epigenetic modifiers mutations in relapsed FLT3mut AML patients, followed by the acquisition of the TP53 and WT1 mutations, whereas F691L represents the classical mutation conferring resistance to all currently available FLT3i. 

FLT3-mut AML patient relapse might be due to FLT3 and its activation of downstream pathways (STAT5, MTOR, JAK). Stromal factors could bypass FLT3 silencing, activating its downstream signaling or stimulating the antiapoptotic protein BCL2 and AXL gene. CXCR4-CCL5 interaction may protect and hide leukemic cells in the bone marrow niche, where cytochromes expressed by stromal cells may also interfere with FLT3i metabolism. Increased fatty acid metabolism in resistant leukemic cells, induced by stromal factors, was also shown to activate cell cycle regulator genes such as CDC7/AURK, with consequent increase of leukemic proliferation.

Interpretation of past clinical trials and post hoc analysis of primary and secondary mechanisms of resistance could guide future personalized treatment plans, tailored to patient populations. 

These approaches could be particularly appealing in patients not eligible for HSCT, but could also be crucial in pursuing cure in the unfavorable context of pre- and post-HSCT R/R disease. Sorafenib maintenance after HSCT has not been approved, but new FLT3i are being investigated in this setting and could likely show greater benefit and safety. Immunotherapy, BCL2, Aurora kinases, Menin, JAK2 inhibitors represent some of the exciting target drugs, investigated in clinical and preclinical trials, which could probably overcome FLT3i resistance and give a breakthrough in the future treatment of FLT3mut AML.

Analysis of quality-of-life-adjusted costs should be performed to guide the choice between combinations of multiple target agents and or sequential pre-emptive treatment of relapse, based on MRD data and relative mutations repertoire.

## Figures and Tables

**Table 1:** List of queries performed for the selection of papers and clinical trials in the different chapters.

Query	Chapter
Midostaurin, Gilteritinib, Quizartinib, Sorafenib and AML and clinical trial (PubMed)	Clinical Trial analyzing FLT3 Target Therapies in AML (2.1)
FLT3 inhibitors and AML and allogeneic HSCT (PubMed)	Maintenance after allogeneic HSCT (2.1.5)
FLT3 inhibitors and AML and mechanisms of resistance (PubMed, last 5 years)	Analysis of Refractory relapsed patients after FLT3 inhibitors exposure (2.2) Mechanisms of resistance, in vitro studies (2.2.1)
FLT3 AML and NPM comutation (PubMed, last 5 years)	Comutation occurrence FLT3/NPM (3.3)
FLT3 AML and IDH comutation (PubMed, last 5 years)	Comutation occurrence FLT3/IDH (3.3)
FLT3 inhibitors and overcoming resistance (PubMed, last 5 years)	Overcoming resistance (2.3)
FLT3 AML and immunotherapy (PubMed, last 5 years)	Immunotherapy (2.3.5)
FLT3 AML and phase I and phase II clinical trial (PubMed, last 5 years)	Phase I trials (2.3.6), Phase II trials (2.3.7)
FLT3mut AML first-line and relapse (Clinicaltrial.gov)	Future directions and ongoing clinical trials (2.3.8)

**Table 2:** FLT3i sensitivities for FLT3 D835Y and F691L mutations.

	FLT3i				
	Midostaurin	Sorafenib	Quizartinib	Gilteritinib	Crenolanib
**ITD**					
**D835Y**					
**F691L**					
**Type**	I	II	II	I	I

Sensitivity: green = sensitive, IC50 ≤ IC50 of FLT3ITD; red = resistant, IC50 > two-fold increase in IC50.

**Table 3:** List of new FLT3i identified in preclinical studies with sensitivities and resistance repertoire.

	Target	Sensible Mutations	Resistant Mutations
Pexidartinib (PLX3397) [62]	FLT3ITD, CKIT, CSFR	FLT3ITD, FLT3 F691L	FLT3 D835Y
Lu50 [63]	FLT3	FLT3ITD, FLT3 F691L, FLT3 D835V	-
NCGC1481 [64,65]	FLT3, IRAK1/4	FLT3D835-V,H,Y, FLT3 K663Q, N841I, R834Q, K429A	-
FF10101 [66]	FLT3	FLT3ITD, FLT3 D835, F691, Y842	-
Compound 67 [67]	FLT3ITD	FLT3ITD, FLT3 D835, F691	-
LAM-003 [68]	HSP 90, KDM6A	FLT3ITD, FLT3 D835, F691	-
LT-171-861 [69]	FLT3	FLT3ITD, FLT3 D854, D835Y, F691L, Y842C	-
Compound 17 [70]	FLT3ITD	FLT3ITD, FLT3 F691L, D835-Y,V FLT3 D835-V,H,Y,	-
Compound 8r [71]	FLT3, CAMKK1, TRKC	FLT3ITD NPOS, W51, FLT3 D835Y, FLT3 F594_R595, FLT3R595_E596, FLT3 Y591_V592	-
Compound 5o [72]	FLT3ITD	FLT3 ITD, D835-V,Y, F691L	-

**Table 4:** Summary of preclinical studies with FLT3i and target agents and their mechanisms of action.

Combinations of FLT3i, FLT3i and Target Agents in Recent Preclinical Studies	
Involved Pathway, Mechanism of Action	Target Drugs
FLT3i combinations	WS6 + Ispinesib + Cabozantinib [73]
MYC-BCL2	BET inhibitors + Quizartinib [74]
PTPN11	Dasatinib + Quizartinib [75]
PI3K-AKT-MTOR/HSP-MEK inhibitors	HSP90-MEK inhibitors Rapamycin [76] LY294002 + Sorafenib [77]
JAK1-CSF2RB–STAT5	Momelotinib + Gilteritinib/Quizartinib [78]
FOXO1- and FOXO3-mediated transactivation of histone deacetylase 8 (HDAC8)→p53 inhibition	HDAC8 inhibitor (22d) + Quizartinib [79]
MDM2 inhibitor	NVP-HDM201 + Midostaurin [80]
AXL inhibitors	DAXL-88-MMAE + Quizartinib [81]
Multitarget agents	
FLT3, AXL, MET, VEGFR, and KIT	Cabozantinib [82]
FLT3, JAK2	Compound 14j [83]
FLT3, Aurora kinases	CCT241736 [84]
FLT3 and AMPKα proteins	Wu-5 [85]
FLT3 and tubulin inhibitor	KX2-391 [86]
FLT3 and Hedgehog signaling—GLI2 inhibition- c-Myc decreasing and p53 increasing	Triptonide [87]

**Table 5:** List of ongoing clinical trials including chemotherapy or Hypomethylating agents andFLT3i.

ClinicalTrial Id.	Drug/Drugs Combination	Phase	Setting
NCT03836209	Gilteritinib+CT vs. Midostaurin+CT	II	DE NOVO FLT3+ AML
NCT04240002	Gilteritinib+CT	I–II	DE NOVO FLT3+ AML
NCT04027309	Gilteritinib+CT vs. Midostaurin+CT	III	DE NOVO FLT3+ AML
NCT05024552	Vyxeos+Gilteritinib	I	RR FLT3+ AML
NCT03735875	Venetoclax+Quizartinib	I	RR FLT3+ AML
NCT03250338	Crenolanib+CT vs. CT	III	RR FLT3+ AML
NCT04140487	Aza+Venetoclax+Gilteritinib	I–II	RR FLT3+ AML
NCT04293562	Vyxeos+/-Gilteritinib vs. SOC	III	DE NOVO FLT3 +/− AML
NCT04047641	Quizartinib+CT	II	RR AML
NCT01892371	Quizartinib + Azacitidine/LDAC	I–II	RR FLT3 +/− AML
NCT04687761	Azacitidine/LDAC+Venetoclax+Quizartinib	I–II	DE NOVO AML (elderly)

**Table 6:** List of ongoing clinical trials including combination of new multitarget agents and FLT3i or hypomethylating agents.

ClinicalTrial Id.	Drug/Drugs Combination	Phase	Setting
NCT05023707	anti-FLT3 CAR-T	I–II	RR FLT3+ AML
NCT04518345	Dubermatinib	I–II	RR FLT3+ AML
NCT05241106	HYML-122	II	RR FLT3+ AML
NCT05010122	ASTX727, Venetoclax, and Gilteritinib	I–II	FLT3+ RR/DE NOVO AML; HR-MDS
NCT04669067	TL-895, KRT-232	I–II	RR TP53wt AML
NCT05028751	Lanraplenib and Gilteritinib	I–II	RR FLT3+ AML
NCT04716114	SKLB1028 vs. salvage	III	RR FLT3+ AML
NCT04842370	PHI-101	I	FLT3+/− AML
NCT05143996	CLN-049	I	RR FLT3+/− AML
NCT03922100	NMS-03592088	II	RR FLT3+ AML; CMML
NCT04827069	Clifutinib Besylate	I	RR FLT3+ AML
NCT03412292	MAX-40279	I	RR FLT3+ AML
NCT04278768	CA-4948 +/-Azacitidine+Venetoclax	I–II	RR FLT3+/− AML
NCT05061147	MAX-40279-01	I–II	RR AML
NCT05279859	ERAS-007/ERAS-601+ Gilteritinib	I–II	RR FLT3+ AML
NCT03513484	Nintedanib	I	RR AML
NCT04477291	CG-806	I	FLT3+/− RR AML

## Abbreviations

AML	Acute Myeloid Leukemia.
FLT3i	FLT3 inhibitors.
FLT3mut	FLT3 mutated.
FLT3ITD	FLT3 internal tandem duplication.
FLT3TKD	Tyrosine kinase domain mutations
JM	Juxtamembrane.
HSCT	Allogeneic Hemopoietic Stem Cell Transplantation.
R/R	Relapsed/refractory.
PDGF	Platelet-derived growth factor.
OS	Overall survival.
EFS	Event free survival.
DFS	Disease free survival.
CCR	Composite complete remission.
Cri	Complete remission with incomplete hematologic recovery.
CRp	Complete remission with incomplete platelet recovery.
VEGF	Vascular endothelial growth factor.
RFS	Relapse free survival.
ORR	Overall Response rate.
MRD	Minimal residual disease.
MRDneg	Negative minimal residual disease.
MRDpos	Positive minimal residual disease.
GVHD	Graft-versus-host disease.
FL	FLT3 ligand.
VAF	Variant allel frequency.
CCT	Conventional chemotherapy.
LIT	Low intensity therapy.
PCR	Polymerase chain reaction.
WES	Whole exome sequencing.
CR	Complete remission.
FGF2	Fibroblast growth factor 2.
CRISPR-Cas9	Unbiased genome-wide clustered regularly interspaced short palindromic repeats.
GLS	Glutaminase.
Rac1	Ras-related C3 botulinum toxin substrate 1.
LSCs	Leukemic stem cells.
OME	Omacetaxine mepesuccinate.
BID	Bis in die.
HMA	Hypomethylating agents.
